# Integrating PrEP in maternal and child health clinics in Kenya: analysis of a service availability and readiness assessment (SARA) survey

**DOI:** 10.3389/frph.2023.1206150

**Published:** 2023-07-06

**Authors:** Sarah Hicks, Felix Abuna, Ben Odhiambo, Julia C. Dettinger, Joseph Sila, George Oketch, Enock Sifuna, Nancy Ngumbau, Laurén Gómez, Grace C. John-Stewart, John Kinuthia, Anjuli D. Wagner

**Affiliations:** ^1^ Department of Epidemiology, University of Washington, Seattle, WA, United States; ^2^ Research and Programs, Kenyatta National Hospital, Nairobi, Kenya; ^3^ Department of Global Health, University of Washington, Seattle, WA, United States; ^4^ Departments of Medicine, University of Washington, Seattle, WA, United States; ^5^ Departments of Pediatrics, University of Washington, Seattle WA, United States

**Keywords:** pre-exposure prophylaxis (PrEP), service readiness and availability, commodities, supervision, pregnancy, postpartum, health facilities (MeSH)

## Abstract

**Background:**

Risk of HIV acquisition is high during pregnancy and postpartum, and pre-exposure prophylaxis (PrEP) is recommended for peripartum populations. Integrating PrEP into maternal and child health (MCH) clinics is feasible and acceptable. Understanding clinics' service availability and readiness is essential for effective scale up.

**Methods:**

The PrEP in Pregnancy, Accelerating Reach and Efficiency study (PrEPARE; NCT04712994) engaged PrEP-experienced facilities previously linked to a programmatic or research study in Western Kenya to document available services and commodities via a modified service availability and readiness assessment (SARA) survey with 20 PrEP tracer items covering: staffing/guidelines, services/equipment, and medicines/commodities. Facilities' prior study engagement occurred between 2017 and 2019; SARA survey data was collected between April 2020 and June 2021. Descriptive statistics were stratified by prior study engagement. ANOVA tests assessed associations between facility characteristics and gaps. Fisher's tests assessed differences in commodity availability and stockouts.

**Results:**

Of the 55 facilities surveyed, 60% had received PrEP training in the last two years, 95% offered PrEP integrated into MCH, and 64% and 78% had both auditory and visual privacy in PrEP and HIV testing service (HTS) delivery spaces, respectively. Supervision frequency was heterogeneous, but 82% had received a supervision visit within 3 months. Availability of commodities was variable and the most commonly unavailable commodities were PrEP in MCH (71% available) and risk assessment screening tool (RAST) and PrEP cards (60% and 75% available, respectively). The number of service and commodity gaps per facility ranged from zero to eight (median: 3; IQR: 2, 5). The most frequent gaps were: PrEP training and risk assessment cards (40% each), lack of privacy in PrEP (36%) and HIV testing services (31%) spaces, PrEP pills in MCH (29%), and PrEP cards (25%). There were no differences in mean number of gaps by county, previous study engagement, or public vs. private status. Level 4 facilities had fewer gaps (mean 2.2) than level 2, 3, and 5 facilities (mean 5.7, 4.5, and 5.3 respectively; *p* < 0.001).

**Conclusions:**

PrEP service availability and readiness was generally high across MCH facilities. However, there is a need for increased frequency of provider training and supportive supervision focused on fidelity. To address key commodity stockouts such as PrEP pills, implementation of electronic logistics management information systems may be needed. Targeting these gaps is essential to effectively scale up integrated PrEP delivery, especially among facilities with limited infrastructure.

## Introduction

HIV incidence among women is high during pregnancy and the postpartum period (1, 2). Women who acquire HIV infections during these periods of elevated risk contribute disproportionately and increasingly to vertical HIV transmission (3–5). Pre-exposure prophylaxis (PrEP) is recommended by both WHO and Kenyan guidelines during pregnancy and postpartum (6–9). Several studies have found that PrEP is safe and effective during pregnancy (10–13). In order to assess effective PrEP delivery for high-risk populations, the PrEP care cascade is used to identify gaps in intervention and program delivery as well as behavioral factors such as HIV risk perception (14, 15). Previous evaluations of PrEP delivery interventions and programs in sub-Saharan Africa have not incorporated data on service and commodity availability (16, 17). Assessing readiness of facilities for high quality PrEP delivery is useful in scale up planning, and previous work has called for the integration of demand, supply, and adherence analysis in HIV prevention program planning with specific interventions such as PrEP (15, 18–20).

PrEP delivery within maternal and child health (MCH) services is feasible and preferable to PrEP provision in HIV care clinics in Kenya (21–24). However, there is suboptimal implementation and integration of PrEP in MCH and family planning (FP) clinics, and MCH/FP clinic-delivered PrEP programming has not yet been systematically scaled up. In order to reduce siloed PrEP delivery for at-risk pregnant and postpartum women, there is a need for enhanced focus on the gaps in service availability and readiness in non-HIV dedicated clinics within the Kenyan health sector (25).

Service availability and readiness assessment (SARA) surveys are useful to track essential commodities and practices by systematically documenting availability of tracer items across facilities to identify strengths and gaps in service provision (26). These surveys may aid in meeting Kenya's strategic health sector goals because many reported barriers to community health services access are at the health facility level (27). The Kenya Harmonized Health Facility Assessment and previous qualitative work with HCWs experienced in PrEP delivery showed that healthcare worker (HCW) shortages, commodity shortages, and a lack of essential amenities impede access to community health services (27, 28). While barriers are understood, the lack of tracking and documenting these barriers at the individual facility level impedes the ability to integrate PrEP in MCH services.

The first component of programs aiming to prevent vertical HIV transmission is preventing HIV acquisition among pregnant women, yet this vital first prong receives little attention in Kenyan policies on vertical transmission (29). In addition to the limited scope of prevention efforts, the monitoring of PrEP service availability and readiness has been incomplete. The most recent national SARA survey was conducted in 2013, prior to the national launch of PrEP in 2017, and this survey found that 60% of facilities in Kenya were providing vertical transmission prevention services (30). However, ART drugs were the sole focus of the HIV commodity assessment, highlighting the need for an updated look at commodity availability following national PrEP scaleup. Integrating PrEP into MCH clinics and reducing vertical HIV transmission will remain a substantial challenge without understanding service and commodity availability in more granular detail.

This analysis comprises the largest sample of facilities with experience delivering PrEP in MCH. As each of these facilities have previously engaged in studies and programs related to HIV prevention for pregnant and postpartum women, we would expect a higher degree of service availability and readiness compared to all facilities across the counties. Identifying gaps in services and readiness after the conclusion of these prior research activities can inform strategic efforts to address these gaps and scale up intervention efforts. This descriptive and exploratory analysis provides a detailed assessment of the items necessary for delivering comprehensive HIV prevention for women at risk of HIV acquisition in the context of MCH services.

## Methods

### Study design

The *PrEP in Pregnancy, Accelerating Reach and Efficiency* (PrEPARE; NCT04712994) study develops, pilots, and evaluates four implementation strategy bundles to optimize PrEP integration and delivery in MCH and FP clinics. This analysis is a cross-sectional evaluation of facility Service Availability and Readiness Assessment (SARA) surveys (26). Data was collected between April 2020 and June 2021, prior to the implementation of strategy bundles.

### Study facilities and prior study engagement

The SARA surveys were completed at facilities from three counties in Kenya: Kisumu, Homa Bay, and Siaya Counties. Each facility had previously participated in a component of the suite of PrEP in pregnancy studies: *PrEP Implementation for Young Women and Adolescents* program (PrIYA) (31), PrIYA Mentorship program (31), and *PrEP Implementation for Mothers in Antenatal Care* study (PrIMA; NCT03070600). A timeline of data collection across these studies is available in Supplementary Figure S1. *PrIYA description*: PrIYA sites integrated PrEP delivery for at-risk adolescent girls and young women attending family planning and pregnant and postpartum women and other women attending maternal and child health clinics in Kenya (31). Women seeking care at these facilities who had tested HIV negative at that visit or within a month, and were willing to receive PrEP counselling were offered PrEP. PrIYA activities were conducted between June 2017 and December 2018; in this programmatically-focused project, there was no specific intervention tested in a comparative design; instead, facilities focused on navigating how to deliver integrated PrEP in MCH and FP clinics in diverse settings. Study staff assisted with program implementation and service delivery during the study period; these additional staff were phased out after the first year (31). *PrIYA Mentorship description:* PrIYA Mentorship site activities were conducted between January and July 2018. There were no study procedures used, but former PrIYA nurses provided in-clinic guidance to existing HCWs at PrIYA Mentorship sites to assist with implementation; study staff were not involved in service delivery for PrIYA mentorship. *PrIMA description*: Finally, PrIMA was a research study that provided additional staff to assist in study activities. The 20 public clinic sites involved in PrIMA were assigned to one of two arms in a cluster randomized trial; pregnant women seeking routine MCH care at these clinics either (a) self-selected into PrEP after receipt of PrEP counseling (Universal arm), or (b) were evaluated for HIV risk via an objective risk-scoring tool and offered HIV self-tests for at-home partner testing (Targeted arm) (32, 33). In the Targeted arm, only individuals determined to be high risk for HIV acquisition were offered PrEP. PrIMA was conducted with study staff between January 2018 and July 2019.

### Ethical approval

All participants provided oral informed consent to participate. This study was approved by the ethical review Committees at the University of Washington and Kenyatta National Hospital.

### Data collection

At each facility, a healthcare worker with experience delivering PrEP to pregnant and postpartum populations was asked to complete the SARA survey; the healthcare workers were purposively selected for higher levels of experience by the study staff who were familiar with the healthcare workers' level of experience at their facility. The SARA survey is a health facility assessment tool developed by the World Health Organization (WHO) and United States Agency for International Development (USAID) (26). A set of tracer items (commodities, clinical practices, and behaviors) is generated for the survey that allows for a systematic measure of facility service availability and readiness in a particular field of healthcare (see Supplementary Table S1 for list of tracer items used in this survey). We adapted standard tracer items from HIV care to be applicable to PrEP delivery, including HIV testing services (HTS). Participants were asked to provide information on facility characteristics (e.g., facility level, urbanicity) and a set of 20 PrEP-delivery-specific tracer items which were categorized as pertaining to staffing and guidelines (e.g., training, supervisory visits), services and equipment (e.g., MCH services, private spaces for PrEP delivery), and medicines and commodities (e.g., HIV rapid test kits, PrEP pills, stockouts in the last month). All data on facility characteristics and the 20 tracer items were self-reported by the healthcare worker who completed the SARA survey for that facility. The surveys were administered online, over the phone, or in-person through REDCap, a secure, online data collection and management software (34).

### Data analysis

Descriptive statistics – including counts and proportions – were calculated to summarize the facility readiness based on the presence of tracer items. In sensitivity analyses, descriptive statistics were stratified by facilities' prior engagement in PrIYA, PrIYA Mentorship, and PrIMA.

A heatmap was generated to identify common gaps across service availability and readiness tracer items for all facilities. Descriptive statistics, including average number of gaps, for each facility are provided. All gaps were coded as binary variables (1 = Yes, 0 = No), unless otherwise specified. Gaps in HTS and PrEP delivery spaces were defined as having no privacy, auditory privacy only, or visual privacy only. Gaps in supervision frequency were defined as not having received a supervisory visit within the last three months, in alignment with the recommendations from the Kenyan Ministry of Health (35). ANOVA tests were used to compare the average total number of gaps between the categories of facilities' county, level (categorization of facilities based on services provided and geographic region served; categorized as levels 1–6) (36), previous study enrollment, and managing authority using an *α*-level of 0.05.

To assess the relationship between current commodity availability and commodity stockouts, Fisher's exact tests were used with an *α*-level of 0.05. Commodities were coded as binary variables (1 = currently available vs. 0 = previously available or no); stockouts within the last month were similarly coded as a binary variable (yes, no).

## Results

### Facility characteristics

A total of 55 health facilities were included in this analysis; descriptive characteristics are included in Table 1 and Supplementary Table S2. The facilities included three dispensaries or clinics (level 2), 15 health centers (level 3), 34 sub-county hospitals or medium private hospitals (level 4), and three county referral hospitals or large private hospitals (level 5). There were no SARA surveys completed at community service centers (level 1) or national referral hospitals (level 6). There were 16 PrIYA facilities, 20 PrIYA Mentorship facilities, and 19 PrIMA facilities; 100% of PrIYA and PrIYA Mentorship facilities were in Kisumu County, while PrIMA facilities were located in Siaya and Homa Bay Counties (53% and 47% respectively). The majority of facilities (91%) were government or public facilities, and nearly all facilities worked with an implementing partner to deliver PrEP (95%). The plurality of facilities were located in rural areas (45%), followed by semi-urban (40%) and urban (15%) areas.

**Table 1:** Characteristics of the health facilities assessed. Kenya, 2020–2021.

	Overall (*N*=55)
County	
Homa Bay	9 (16%)
Siaya	10 (18%)
Kisumu	36 (66%)
Facility level	
2 – Dispensary or clinic	3 (6%)
3 – Health center	15 (27%)
4 – Sub-county hospital or private medium hospital	34 (62%)
5 – County referral hospital or large private hospital	3 (6%)
Managing authority	
Government/public	50 (91%)
Mission/faith-based	4 (7%)
Private-for-profit	1 (2%)
Implementing partner	
Yes	52 (95%)
Urbanicity	
Urban	8 (15%)
Semi-urban	22 (40%)
Rural	25 (45%)

### SARA survey tracer items

**Figure 1:**
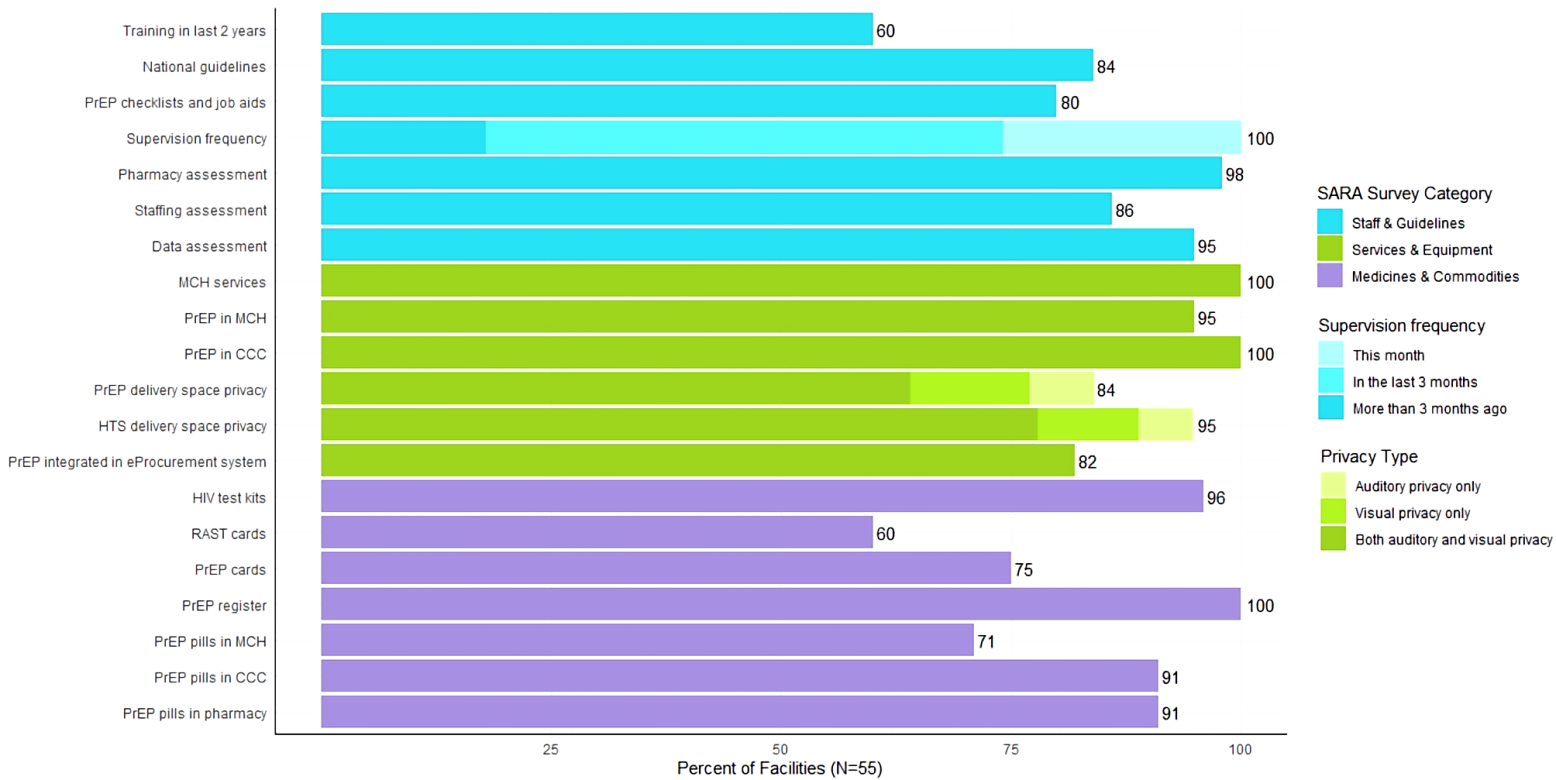
Percentage of facilities that have tracer items for PrEP delivery. Kenya, 2020–2021.

Twenty items were assessed in the SARA surveys to determine the current availability of staff and guidelines, services and equipment, and medicines and commodities (Figure 1). Sixty percent of facilities had received PrEP training within the past 2 years; 84% had national guidelines available at the site, and 80% had PrEP checklists and job aids at the site. PrEP training included trainings conducted by study staff, Ministry of Health officials, or other implementing partners. The majority (82%) of facilities had received a supervisory visit within the last three months, which included assessments of staffing, data, and pharmacy supplies. However, fewer (26%) had received a supervision visit within the last month.

All facilities offered MCH services and provided PrEP in HIV care clinics, and nearly all facilities still provided PrEP in MCH (95%). However, there were gaps identified in the amount of privacy provided in PrEP and HTS delivery spaces. Approximately 64% and 78% of facilities had both auditory and visual privacy for these delivery spaces, respectively. Integration of PrEP ordering into the eProcurement system was reasonably high at 82%.

Medicine and commodity availability was more varied across facilities. Most facilities reported availability of HIV test kits (96%), PrEP registers (100%), and PrEP pills in HIV care clinics (91%) and pharmacies (91%). However, availability of risk assessment screening tool (RAST) cards, PrEP cards, and PrEP pills in MCH clinics was lower (60%, 75%, and 71%, respectively).

### Sensitivity analysis stratified by prior study engagement

In order to characterize the differences in infrastructure that might distinguish facilities chosen for research studies vs. more typical facilities, we conducted sensitivity analyses stratified by prior study engagement (Supplementary Figure S2). Of note, facilities selected as trial sites (PrIMA) had greater visual and auditory privacy than facilities selected as demonstration project sites (PrIYA) or expanded capacity-building sites (PrIYA mentorship). Similarly, facilities selected as trial and demonstration project sites were more likely to have eProcurement systems for PrEP than capacity-building sites (PrIYA mentorship). There were no meaningful differences between facilities selected as trial, demonstration project, or capacity-building sites in terms of availability of HIV test kits, PrEP registers, PrEP pills in HIV care clinic and pharmacy.

### Frequency and heatmap of gaps

Across the 20 tracer items assessed, the number of facilities reporting a gap ranged from 22 facilities having not received a PrEP training in the last two years to zero facilities having a gap for having a PrEP register, offering PrEP in HIV care clinics, and offering MCH services (Supplementary Table S1).

Across the 55 facilities surveyed, the number of gaps ranged from zero to eight (median: 3; IQR: 2, 5). In an exploratory analysis, there was a significant difference in the total number of gaps based on facility level. The level 4 facilities (sub-county hospitals or private medium hospitals) had an average of 2.9 gaps compared to an average of 5.3, 3.7, and 5.3 gaps among level 2, 3, and 5 facilities respectively (dispensaries or clinics, health centers, and county referral hospital or large private hospitals respectively) (*p* < 0.001). There were no significant differences in the average number of gaps between facilities in different counties, by previous study enrollment, or by managing authority.

### Concordance between current commodity availability and stockouts in the last month

Within the SARA survey, six commodities were measured in terms of current availability and history of stockout; we compared the two measures to determine the level of agreement between them by assessing two categories of concordance (stockout in the last month & commodity not available currently; no stockout in the last month & commodity available currently) and discordance (expected: stockout in the last month & commodity available currently; unexpected: no stockout in the last month & commodity not available currently). Across the six tracer items assessed, there was generally high concordance between reporting no stockout in the last month and current availability of the commodity, ranging from 45.5% for RAST cards to 88.9% for PrEP registers (Supplementary Table S3). Approximately 10%–15% of facilities reported a stockout in the last month and that the commodity was currently available, although this discordance between measures was expected due to the potential for restocking supplies over a month-long period. However, substantial discordance was observed in reports of availability for RAST cards and PrEP pills in MCH; for these commodities, 18.2% and 16.4% of facilities respectively reported no stockouts in the last month but that these commodities were currently unavailable. The Fisher's exact test could not be performed for the PrEP register (commodity was available at all facilities) or PrEP pills in HIV care clinics (no facilities reported stockouts in the last month and commodity currently unavailable). The four remaining commodities did have statistically significant associations, demonstrating non-random classification of commodity availability by the two measures.

## Discussion

In the present study, we observed generally high service availability and readiness across facilities in three Kenyan counties. Lack of PrEP training and RAST cards were the most common gaps across facilities, followed by PrEP and HTS delivery space privacy, PrEP pills in MCH, and PrEP cards. Differences in infrastructure, but not commodities, between facilities selected for trial, demonstration project, and capacity-building activities reveal insights for PrEP scale-up in MCH clinics.

We observed that HCW training on PrEP delivery was one of the most common gaps. There were fewer gaps in supervision frequency in the last three months, but substantially fewer facilities whose last supervisory visit occurred in the past month. This survey question did not differentiate between trainings conducted by study staff, Ministry of Health officials, or other implementing partners, limiting inference for future scale-up efforts. Provider knowledge of PrEP is necessary for PrEP service scale-up (37–39). Qualitative work among HCWs delivering PrEP in Tanzania highlighted the need for repeat trainings on PrEP, and previous work in Kenya found that repeated encounters with standardized patient actors improved provider counseling and adherence to national PrEP guidelines (38, 40). One study showed that, following in-service trainings among HCWs in a variety of fields, there was a reduction in outcome-associated effectiveness each month after training, highlighting the waning impact of training over time (41). In light of this finding, measuring receipt of provider training within the last two years may overestimate the readiness of facilities to provide PrEP services in MCH with high fidelity. A shift to providing refresher trainings and providing supportive supervision at the intended frequency of 3-monthly for PrEP delivery teams in MCH may be needed to sustain quality care.

While MCH and PrEP services were offered across most facilities, privacy was lacking for both PrEP and HTS delivery spaces. Stigma remains a major concern during pregnancy and postpartum and contributes to avoidance of HIV prevention health services (21, 22, 42, 43). For individuals not living with HIV, there is an aversion to being seen receiving services at clinics associated with HIV for fear of being stigmatized. Additionally, privacy is essential in PrEP counseling sessions, which include inherently sensitive questions regarding sexual history (44, 45). Without providing adequate privacy for HTS and PrEP delivery, it will be challenging to scale-up integration of PrEP in MCH in order to reach women with greatest need (46, 47). In the literature, the majority of stigma-reduction interventions focus on reducing stigma among HCWs or reducing internalized stigma among people living with HIV; these methods include trainings for HCWs and popular opinion leaders, group education and trainings including people living with HIV, restructuring facility anti-discrimination policies, and rarely, social media campaigns (48–51). There is a need for stigma-reduction interventions that target people not living with HIV that will enable them to take full advantage of HIV prevention services.

Additionally, a relatively large proportion of facilities selected for capacity-building activities (PrIYA mentorship facilities), which were commonly located in rural areas, did not have PrEP ordering integrated into the eProcurement system. Use of electronic record-keeping in logistics management information systems (LMIS) increases the accuracy of commodity supply records and reduces lead time for resupply (52, 53). Previous work showed that rural health facilities can reduce the likelihood of commodity stockouts up to 64% when using an electronic LMIS in conjunction with daily updating in the LMIS system (54). Increasing the use of electronic LMIS, particularly in rural health facilities, may be a useful intervention to reduce stockouts and effectively integrate PrEP in MCH.

We observed low availability of RAST and PrEP cards, as well as PrEP pills in MCH. Study staff noted that in Kenyan clinics delivering MCH-integrated PrEP dispensing, MCH clinics are given a certain supply of medication from the central PrEP pill supply manager (either in pharmacy or HIV care clinic); when there is risk of PrEP stockouts in the facility, the MCH commodities are reallocated to the HIV care clinic pharmacy. This could explain why we observed that more facilities did not have PrEP pills in MCH compared to the HIV care clinic. Study staff also noted that during the period of data collection, many facilities were transitioning from paper-based medical records to electronic medical records (EMR), eliminating the need for paper commodities. While paper commodities were less frequently available, this may not have as substantial an impact on readiness to provide PrEP in MCH as previously thought. Surveys conducted with HCWs at the facilities during the data collection period noted that paper commodity stockouts had little to no impact on their ability to implement PrEP in MCH (*Hicks et al, under review*). As there is currently no standard for the use of paper vs. EMR, future SARA assessments of PrEP delivery in MCH should include both paper and EMR tracer items.

We found that sub-county hospitals or private medium hospitals had fewer gaps compared to the other facility levels included in this analysis. The 2013 Kenya SARA mapping survey found that primary care facilities (Tier 2) had the highest HIV service readiness index score at 78% compared to community (Tier 1; 67%), county (Tier 3; 74%), and national level facilities (Tier 4; 52%) (30). However, dispensaries, clinics, health centers, and sub-county hospitals are included in the Tier 2 definitions from Kenya's Health Policy (30). The additional disaggregation of facility types in this analysis highlights disparities in facility level readiness that will be useful in targeting interventions to improve service readiness for PrEP scale-up and integration in MCH services.

While we observed differences between facilities selected for trial (PrIMA), demonstration project (PrIYA), and capacity-building (PrIYA mentorship) activities, we do not believe that facility engagement in research studies led to higher levels of availability and readiness. Facilities that are selected for research may be more likely to have higher baseline service availability and readiness, which are then supplemented by additional resources and staff provided by the studies. For example, the PrIYA and PrIMA studies selected facilities based on higher patient volumes and assessments of infrastructure readiness. As we look towards scale-up of PrEP integration approaches, we need to be cognizant of these differences and prepare for potentially greater resource gaps among facilities that have not been involved in previous research activities, due to either lower client volumes or organizational readiness.

Generally, we observed concordance between current commodity availability and stockouts within the last month. There was a relatively high proportion of facilities reporting expected concordance – the absence of stockouts across both measures and the presence of stockouts by both measures. However, a substantial proportion of facilities provided conflicting responses. While it is possible and expected that there might be a stockout in the past month but not at present, it is not possible for there to be a stockout at present but not in the last month. However, it is important to note that stockout questions may have been interpreted to mean the last full calendar month which could exclude the present day. While this question was intended to reflect stockouts over the past 30 days including today, misinterpretations may have led to data reporting inconsistencies. Literature on commodity availability across several health topics have included measures of stockouts over varying time periods; the WHO SARA reference manual also includes measures of both current availability and past stockouts for the same commodities (55–58). These findings suggest that both measures should be included in future SARA surveys to avoid underestimation of commodity stockouts.

This study emphasizes HIV prevention services among women not living with HIV in MCH, addressing a gap in academic literature and national vertical transmission prevention programming. We were able to take a facility-specific view of service availability and readiness, enabling the identification of gaps by facility characteristics and prior engagement in studies focused on PrEP integration and delivery within MCH. The more comprehensive list of tracer items enhances our understanding of where service provision and readiness is lacking across facilities in order to target interventions that will assist in integrated PrEP delivery scale up. These study strengths shed light on how to target effective HIV prevention services for this unique population.

However, this study does have several limitations. First, we did not assess provider knowledge of PrEP initiation or continuation guidelines. Previous work from sub-Saharan Africa has shown that poor clinical knowledge has a greater impact on readiness to provide services than either commodity availability or HCW absenteeism (59). While the lack of training was identified in our analysis, we may be missing a key indicator for readiness by not measuring provider knowledge. Second, our sample primarily consisted of Level 3 and 4 facilities that are part of the government or public sector; there is limited generalizability to the private sector or other forms of managing authorities. Third, the survey was completed by a single HCW at each facility who may be subject to recall bias or lack of familiarity with certain components of the survey; we did not collect individual-level data about the healthcare workers, so we are unable to verify the representativeness of these participants and their facilities compared to other facilities in the region. However, this sample reflected all of the facilities in the region with experience delivering PrEP in MCH through the 3 mentioned projects. Additionally, there was no direct observation from study staff as is ideal in SARA surveys, especially for commodities, due to COVID-19 restrictions on facility access. The use of self-report data may be subject to recall bias. Finally, there was differential time since facilities were engaged in PrIYA, PrIYA mentorship, and PRIMA, so readiness may have waned due to staff rotations or other factors outside the control of study staff.

## Conclusions

This study sought to identify strengths and gaps in service availability and readiness across Kenyan health facilities that are integrating PrEP delivery into MCH services. There are overarching gaps that need to be addressed for effective scale-up of PrEP integration in MCH, particularly among dispensaries, clinics, health centers, and county-level hospitals. PrEP training for HCWs needs to be more frequently implemented in addition to supportive supervision focused on fidelity. HTS and PrEP delivery spaces must provide adequate auditory and visual privacy to reduce stigmatization and facilitate PrEP uptake. Although paper commodities were lacking, utilization of EMRs may offset this need for effective PrEP integration. However, PrEP pill stockouts in MCH needs to be addressed, potentially through electronic LMIS and daily updating of stock supplies. As investigators typically select facilities with high client volumes and adequate infrastructure for study engagement, there is a need to consider and account for resource differences when scaling up PrEP delivery strategies, particularly in facilities with limited infrastructure and support.

## Data Availability

The raw data supporting the conclusions of this article will be made available by the authors, without undue reservation.

## Supplementary material

SUPPLEMENTARY FIGURE S1

Timeline and descriptions of previous study engagement. Kenya, 2020–2021.

SUPPLEMENTARY FIGURE S2

Percentage of facilities that have tracer items for PrEP delivery stratified by previous study engagement. Kenya, 2020–2021.

SUPPLEMENTARY TABLE S1

Heatmap of facility tracer items. Kenya, 2020–2021.

SUPPLEMENTARY TABLE S2

Characteristics of the health facilities assessed, overall and stratified by previous study engagement. Kenya, 2020–2021.

SUPPLEMENTARY TABLE S3

Fisher's exact tests of commodities available now vs stockouts in the last month. Kenya, 2020–2021.
