# Supplementary figures and images for: Integrating PrEP in maternal and child health clinics in Kenya: analysis of a service availability and readiness assessment (SARA) survey
Source: Front Reprod Health. 2023 Jul 6;5:1206150. doi: 10.3389/frph.2023.1206150 (PMC10359145; doi:10.3389/frph.2023.1206150)

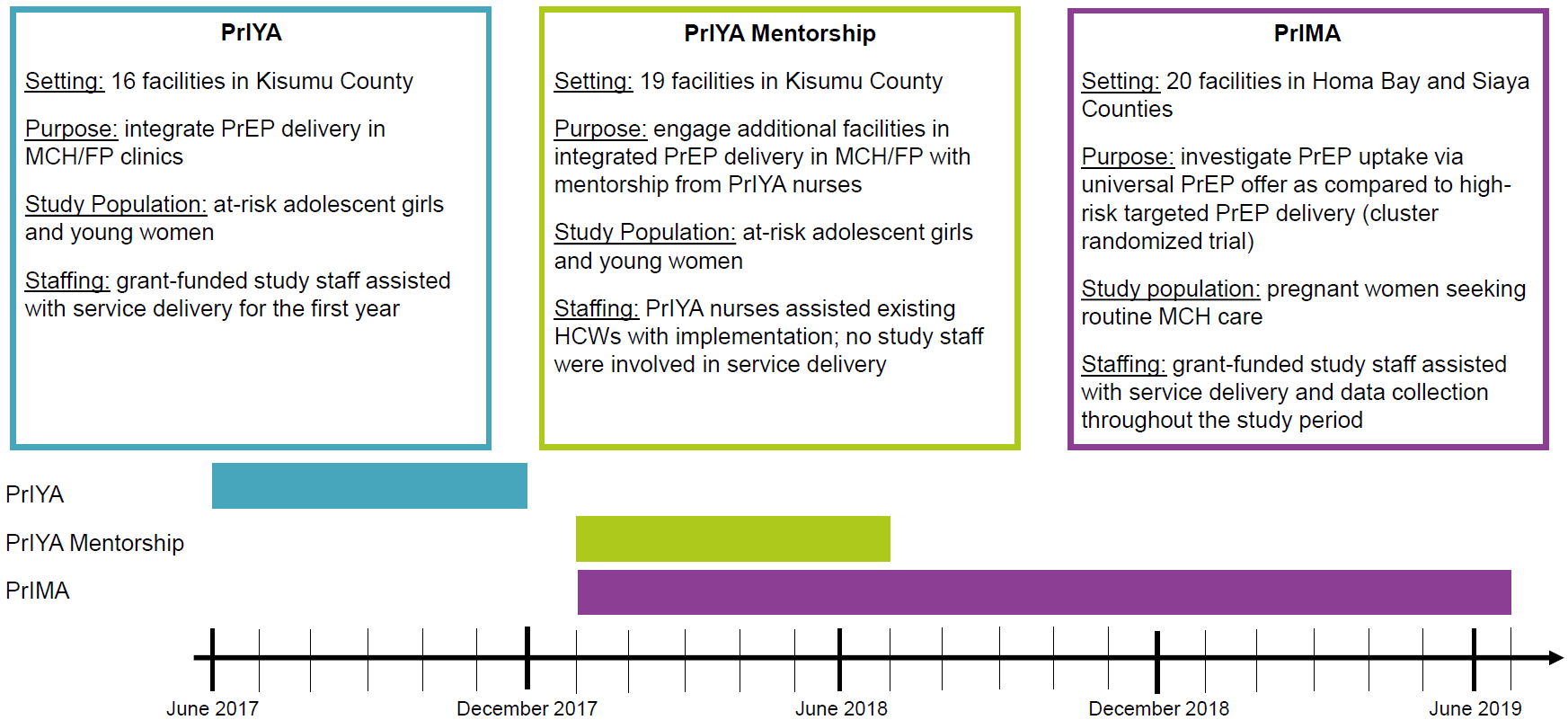

Supplement: Supplementary file 1 [file Image1.tif]

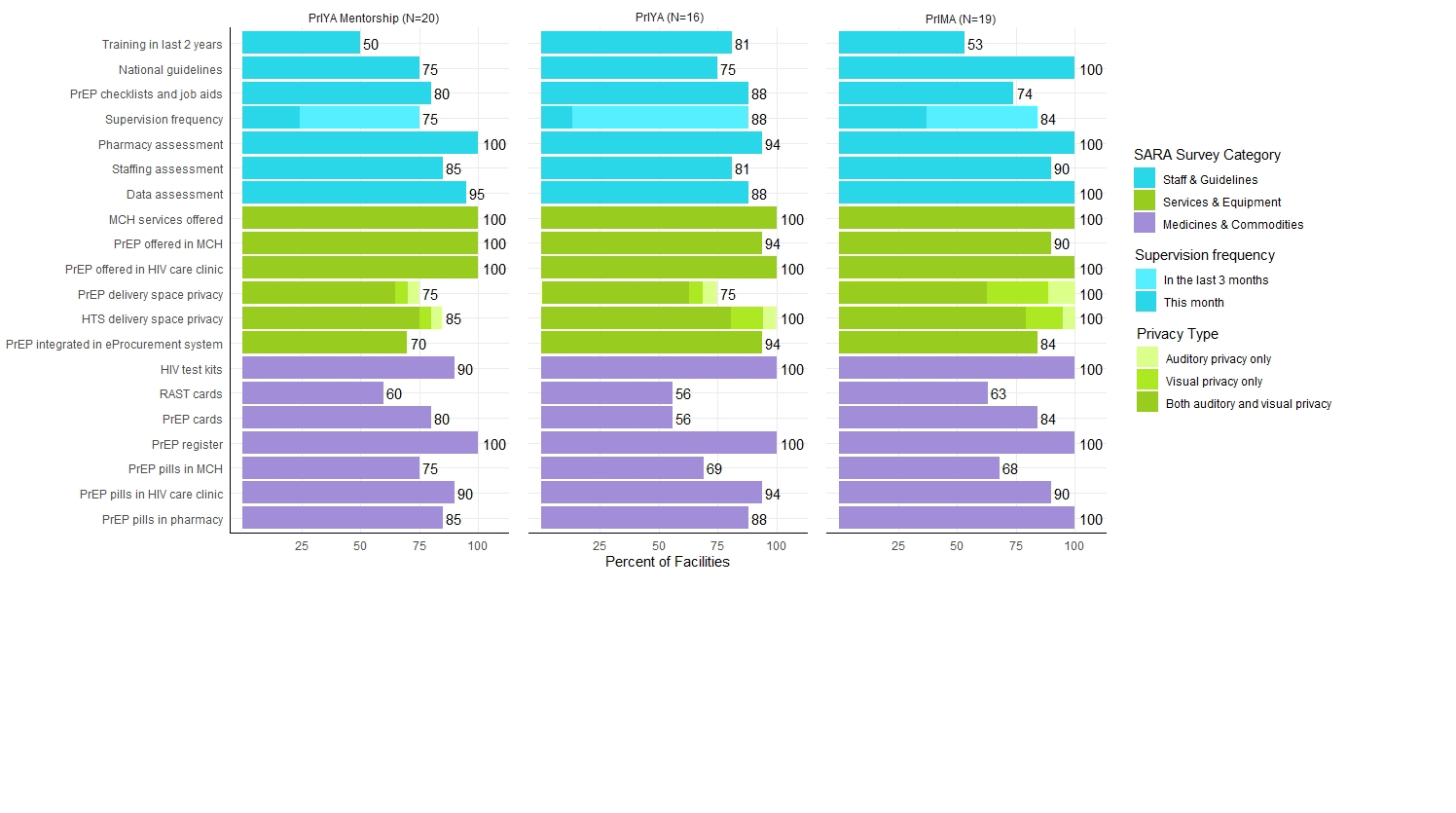

Supplement: Supplementary file 2 [file Image2.tiff]
