# Supplementary material for: Integrating PrEP in maternal and child health clinics in Kenya: analysis of a service availability and readiness assessment (SARA) survey
Source: Front Reprod Health. 2023 Jul 6;5:1206150. doi: 10.3389/frph.2023.1206150 (PMC10359145; doi:10.3389/frph.2023.1206150)
Supplement: Supplementary file 3 [file Table1.pdf]

Facility ID	County	Facility Level	Previous Study Enrollment	Managing Authority	Did not receive PrEP training in last 2 years	RAST cards unavailable at facility	PrEP delivery space lacks both auditory and visual privacy	HIV test delivery space lacks both auditory and visual privacy	PrEP pills in MCH unavailable at facility	PrEP cards unavailable at facility	Did not receive a supervisory visit within the last three months	PrEP checklists and job aids are not posted	PrEP ordering is not integrated into eProcure system	National PrEP guidelines are not posted	Last supervisory visit did not assess staffing	PrEP pills in HIV care clinic unavailable at facility	PrEP pills in pharmacy unavailable at facility	PrEP not offered in MCH clinics	HIV rapid test kits unavailable at facility	Last supervisory visit did not assess data quality	Last supervisory visit did not assess pharmacy	MCH services not offered	PrEP not offered in HIV care clinic	PrEP register unavailable at facility	Total number of gaps at each facility
1	Kisumu	3 - Health Centre	PrIYA Mentorship	Government/public	1	1	1	1	1	1	1	0	0	0	1	0	0	0	0	0	0	0	0	0	8
2	Kisumu	2 - Dispensary or clinic	PrIYA Mentorship	Government/public	1	1	1	1	0	1	1	0	1	1	0	0	0	0	0	0	0	0	0	0	8
3	Kisumu	5 - County referral hospital or large private hospital	PrIYA	Government/public	1	1	1	1	0	0	1	0	1	0	1	0	0	0	0	1	0	0	0	0	8
4	Kisumu	4 - Sub-county hospital or medium private hospital	PrIYA	Government/public	0	0	1	1	1	0	0	1	0	0	0	0	1	1	0	1	0	0	0	0	7
5	Kisumu	3 - Health Centre	PrIYA	Government/public	0	1	1	1	1	0	0	1	0	1	1	0	0	0	0	0	0	0	0	0	7
6	Kisumu	3 - Health Centre	PrIYA Mentorship	Government/public	1	0	1	1	0	0	0	1	0	1	0	0	1	0	1	0	0	0	0	0	7
7	Siaya	3 - Health Centre	PrIMA	Government/public	1	1	1	0	1	0	0	1	0	0	1	0	0	1	0	0	0	0	0	0	7
8	Siaya	4 - Sub-county hospital or medium private hospital	PrIMA	Government/public	0	1	1	1	1	1	0	1	0	0	0	0	0	1	0	0	0	0	0	0	7
9	Homa Bay	4 - Sub-county hospital or medium private hospital	PrIMA	Government/public	0	1	1	1	1	1	0	1	0	0	0	0	0	0	0	0	0	0	0	0	6
10	Kisumu	2 - Dispensary or clinic	PrIYA Mentorship	Government/public	1	1	0	0	0	1	1	0	1	0	0	0	1	0	0	0	0	0	0	0	6
11	Kisumu	3 - Health Centre	PrIYA Mentorship	Government/public	1	0	0	0	1	0	0	0	0	1	1	0	1	0	0	0	0	0	0	0	5
12	Siaya	3 - Health Centre	PrIMA	Government/public	1	1	1	1	0	0	0	0	1	0	0	0	0	0	0	0	0	0	0	0	5
13	Siaya	5 - County referral hospital or large private hospital	PrIMA	Government/public	0	1	1	NA	1	0	1	0	1	0	0	0	0	0	0	0	0	0	0	0	5
14	Kisumu	3 - Health Centre	PrIYA	Mission/faith-based	1	1	0	0	1	0	0	0	0	0	0	1	1	0	0	0	0	0	0	0	5
15	Kisumu	3 - Health Centre	PrIYA Mentorship	Government/public	0	1	0	0	1	0	0	0	0	1	0	1	0	0	1	0	0	0	0	0	5
16	Kisumu	4 - Sub-county hospital or medium private hospital	PrIYA	Government/public	0	0	1	1	0	1	0	0	0	1	0	0	0	0	0	0	1	0	0	0	5
17	Kisumu	3 - Health Centre	PrIYA Mentorship	Government/public	0	1	1	1	0	0	0	1	0	0	0	0	0	0	0	0	0	0	0	0	4
18	Kisumu	3 - Health Centre	PrIYA	Government/public	1	1	0	0	0	1	0	0	0	1	0	0	0	0	0	0	0	0	0	0	4
19	Kisumu	3 - Health Centre	PrIYA Mentorship	Government/public	0	0	0	0	0	0	1	0	1	0	1	0	0	0	0	0	0	0	0	0	3
20	Kisumu	4 - Sub-county hospital or medium private hospital	PrIYA Mentorship	Government/public	0	0	0	0	1	0	1	0	0	0	0	1	0	0	0	0	0	0	0	0	3
21	Kisumu	4 - Sub-county hospital or medium private hospital	PrIYA Mentorship	Government/public	1	0	0	0	0	1	0	0	1	0	0	0	0	0	0	0	0	0	0	0	3
22	Homa Bay	5 - County referral hospital or large private hospital	PrIMA	Government/public	1	0	1	1	0	0	0	0	0	0	0	0	0	0	0	0	0	0	0	0	3
23	Kisumu	2 - Dispensary or clinic	PrIYA Mentorship	Government/public	0	1	0	0	0	0	0	1	0	1	0	0	0	0	0	0	0	0	0	0	3
24	Kisumu	4 - Sub-county hospital or medium private hospital	PrIYA Mentorship	Government/public	1	0	1	1	0	0	0	0	0	0	0	0	0	0	0	0	0	0	0	0	3
25	Kisumu	4 - Sub-county hospital or medium private hospital	PrIYA Mentorship	Government/public	0	0	1	1	0	0	0	0	1	0	0	0	0	0	0	0	0	0	0	0	3
26	Homa Bay	4 - Sub-county hospital or medium private hospital	PrIMA	Government/public	1	0	0	0	1	0	0	0	0	0	0	1	0	0	0	0	0	0	0	0	3
27	Kisumu	4 - Sub-county hospital or medium private hospital	PrIYA	Government/public	0	1	0	0	1	1	0	0	0	0	0	0	0	0	0	0	0	0	0	0	3
28	Siaya	4 - Sub-county hospital or medium private hospital	PrIMA	Government/public	1	0	0	0	1	0	0	0	1	0	0	0	0	0	0	0	0	0	0	0	3
29	Homa Bay	4 - Sub-county hospital or medium private hospital	PrIMA	Government/public	1	1	0	0	0	0	0	0	0	0	1	0	0	0	0	0	0	0	0	0	3
30	Siaya	3 - Health Centre	PrIMA	Government/public	1	0	0	0	0	0	0	0	0	0	0	1	0	0	0	0	0	0	0	0	2
31	Homa Bay	4 - Sub-county hospital or medium private hospital	PrIMA	Government/public	0	1	0	0	0	1	0	0	0	0	0	0	0	0	0	0	0	0	0	0	2
32	Siaya	4 - Sub-county hospital or medium private hospital	PrIMA	Government/public	0	0	1	1	0	0	0	0	0	0	0	0	0	0	0	0	0	0	0	0	2
33	Kisumu	4 - Sub-county hospital or medium private hospital	PrIYA Mentorship	Government/public	0	0	1	1	0	0	0	0	0	0	0	0	0	0	0	NA	0	0	0	0	2
34	Homa Bay	4 - Sub-county hospital or medium private hospital	PrIMA	Government/public	1	0	0	0	0	0	0	1	0	0	0	0	0	0	0	0	0	0	0	0	2
35	Kisumu	3 - Health Centre	PrIYA	Mission/faith-based	0	0	0	0	0	0	1	0	0	0	1	0	0	0	0	0	0	0	0	0	2
36	Kisumu	4 - Sub-county hospital or medium private hospital	PrIYA	Mission/faith-based	0	0	0	0	0	1	0	0	0	1	0	0	0	0	0	0	0	0	0	0	2
37	Kisumu	4 - Sub-county hospital or medium private hospital	PrIYA	Government/public	0	0	1	0	1	0	0	0	0	0	0	0	0	0	0	0	0	0	0	0	2
38	Kisumu	4 - Sub-county hospital or medium private hospital	PrIYA Mentorship	Government/public	0	0	0	0	1	0	0	0	1	0	0	0	0	0	0	0	0	0	0	0	2
39	Kisumu	4 - Sub-county hospital or medium private hospital	PrIYA Mentorship	Government/public	1	1	0	NA	0	0	0	0	0	0	0	0	0	0	0	0	0	0	0	0	2
40	Kisumu	4 - Sub-county hospital or medium private hospital	PrIYA	Government/public	0	0	1	1	0	0	0	0	0	0	0	0	0	0	0	0	0	0	0	0	2
41	Kisumu	3 - Health Centre	PrIYA Mentorship	Government/public	1	1	0	0	0	0	0	0	0	0	0	0	0	0	0	0	0	0	0	0	2
42	Homa Bay	4 - Sub-county hospital or medium private hospital	PrIMA	Government/public	1	0	0	0	0	0	0	1	0	0	0	0	0	0	0	0	0	0	0	0	2
43	Kisumu	4 - Sub-county hospital or medium private hospital	PrIYA	Private-for-profit	0	0	0	0	0	1	0	0	0	0	0	0	0	0	0	0	0	0	0	0	1
44	Homa Bay	4 - Sub-county hospital or medium private hospital	PrIMA	Government/public	0	0	0	NA	0	0	1	0	0	0	0	0	0	0	0	0	0	0	0	0	1
45	Kisumu	4 - Sub-county hospital or medium private hospital	PrIYA	Government/public	0	0	0	NA	0	1	0	0	0	0	0	0	0	0	0	0	0	0	0	0	1
46	Kisumu	4 - Sub-county hospital or medium private hospital	PrIYA	Government/public	0	0	0	0	0	1	0	0	0	0	0	0	0	0	0	0	0	0	0	0	1
47	Kisumu	3 - Health Centre	PrIYA Mentorship	Government/public	1	0	0	0	0	0	0	0	0	0	0	0	0	0	0	0	0	0	0	0	1
48	Siaya	4 - Sub-county hospital or medium private hospital	PrIMA	Government/public	0	0	0	0	0	0	1	0	0	0	0	0	0	0	0	0	0	0	0	0	1
49	Kisumu	4 - Sub-county hospital or medium private hospital	PrIYA	Mission/faith-based	0	1	0	0	0	0	0	0	0	0	0	0	0	0	0	0	0	0	0	0	1
50	Kisumu	4 - Sub-county hospital or medium private hospital	PrIYA	Government/public	0	1	0	0	0	0	0	0	0	0	0	0	0	0	0	0	0	0	0	0	1
51	Kisumu	4 - Sub-county hospital or medium private hospital	PrIYA Mentorship	Government/public	0	0	0	0	0	0	0	0	0	0	0	0	0	0	0	0	0	0	0	0	0
52	Homa Bay	4 - Sub-county hospital or medium private hospital	PrIMA	Government/public	0	0	0	0	0	0	0	0	0	0	0	0	0	0	0	0	0	0	0	0	0
53	Siaya	4 - Sub-county hospital or medium private hospital	PrIMA	Government/public	0	0	0	0	0	0	0	0	0	0	0	0	0	0	0	0	0	0	0	0	0
54	Siaya	4 - Sub-county hospital or medium private hospital	PrIMA	Government/public	0	0	0	0	0	0	0	0	0	0	0	0	0	0	0	0	0	0	0	0	0
55	Kisumu	4 - Sub-county hospital or medium private hospital	PrIYA Mentorship	Government/public	0	0	0	0	0	0	0	NA	0	0	0	0	0	0	0	0	0	0	0	0	0
Column rows					23	22	20	17	16	14	10	10	10	9	8	5	5	3	2	2	1	0	0	0	176
