# Supplementary material for: Integrating PrEP in maternal and child health clinics in Kenya: analysis of a service availability and readiness assessment (SARA) survey
Source: Front Reprod Health. 2023 Jul 6;5:1206150. doi: 10.3389/frph.2023.1206150 (PMC10359145; doi:10.3389/frph.2023.1206150)
Supplement: Supplementary file 4 [file Table2.docx]

Supplementary Table 2: Characteristics of the health facilities assessed, overall and stratified by previous study engagement. Kenya, 2020-2021

|  | PrIYA Mentorship  (N=20) | PrIYA  (N=16) | PrIMA  (N=19) | Overall (N=55) |
| --- | --- | --- | --- | --- |
| County |  |  |  |  |
| Homa Bay | 0 (0%) | 0 (0%) | 9 (47%) | 9 (16%) |
| Siaya | 0 (0%) | 0 (0%) | 10 (53%) | 10 (18%) |
| Kisumu | 20 (100%) | 16 (100%) | 0 (0%) | 36 (66%) |
| Facility Level |  |  |  |  |
| 2 – Dispensary or clinic | 3 (15%) | 0 (0%) | 0 (0%) | 3 (6%) |
| 3 – Health center | 8 (40%) | 4 (25%) | 3 (16%) | 15 (27%) |
| 4 – Sub-county hospital or private medium hospital | 9 (45%) | 11 (69%) | 14 (74%) | 34 (62%) |
| 5 – County referral hospital or large private hospital | 0 (0%) | 1 (6%) | 2 (11%) | 3 (6%) |
| Managing Authority |  |  |  |  |
| Government/public | 20 (100%) | 11 (69%) | 19 (100%) | 50 (91%) |
| Mission/faith-based | 0 (0%) | 4 (25%) | 0 (0%) | 4 (7%) |
| Private-for-profit | 0 (0%) | 1 (6%) | 0 (0%) | 1 (2%) |
| Implementing Partner |  |  |  |  |
| Yes | 18 (90%) | 15 (94%) | 19 (100%) | 52 (95%) |
| Urbanicity |  |  |  |  |
| Urban | 2 (10%) | 3 (19%) | 3 (16%) | 8 (15%) |
| Semi-urban | 5 (25%) | 9 (56%) | 8 (42%) | 22 (40%) |
| Rural | 13 (65%) | 4 (25%) | 8 (42%) | 25 (45%) |
