# Supplementary material for: Integrating PrEP in maternal and child health clinics in Kenya: analysis of a service availability and readiness assessment (SARA) survey
Source: Front Reprod Health. 2023 Jul 6;5:1206150. doi: 10.3389/frph.2023.1206150 (PMC10359145; doi:10.3389/frph.2023.1206150)
Supplement: Supplementary file 5 [file Table3.docx]

Supplementary Table 3: Fisher’s exact tests of commodities available now vs stockouts in the last month. Kenya, 2020-2021.

| Commodity | Concordant: Stockout in the last month & commodity not available currently | Concordant: No stockout in the last month & commodity available currently | Expected discrepant: Stockout in the last month & commodity available currently | Unexpected discrepant: No stockout in the last month & commodity not available currently | Fisher’s exact test p-value |
| --- | --- | --- | --- | --- | --- |
| RAST cards | 21.8% | 45.5% | 14.5% | 18.2% | 0.044 |
| PrEP cards | 20.0% | 60.0% | 14.5% | 5.5% | <0.001 |
| PrEP pills in MCH | 12.7% | 60.0% | 10.9% | 16.4% | 0.037 |
| PrEP pills in HIV care clinics | 0.0% | 76.4% | 14.5% | 9.1% | - |
| PrEP pills in pharmacy | 5.5% | 78.2% | 12.7% | 3.6% | 0.037 |
| PrEP register* | 0.0% | 88.9% | 11.1% | 0.0% | - |

**PrEP register was available at all facilities*
